# Supplementary figures and images for: Genome Sequencing of Pantoea agglomerans C1 Provides Insights into Molecular and Genetic Mechanisms of Plant Growth-Promotion and Tolerance to Heavy Metals
Source: Microorganisms. 2020 Jan 22;8(2):153. doi: 10.3390/microorganisms8020153 (PMC7074716; doi:10.3390/microorganisms8020153)

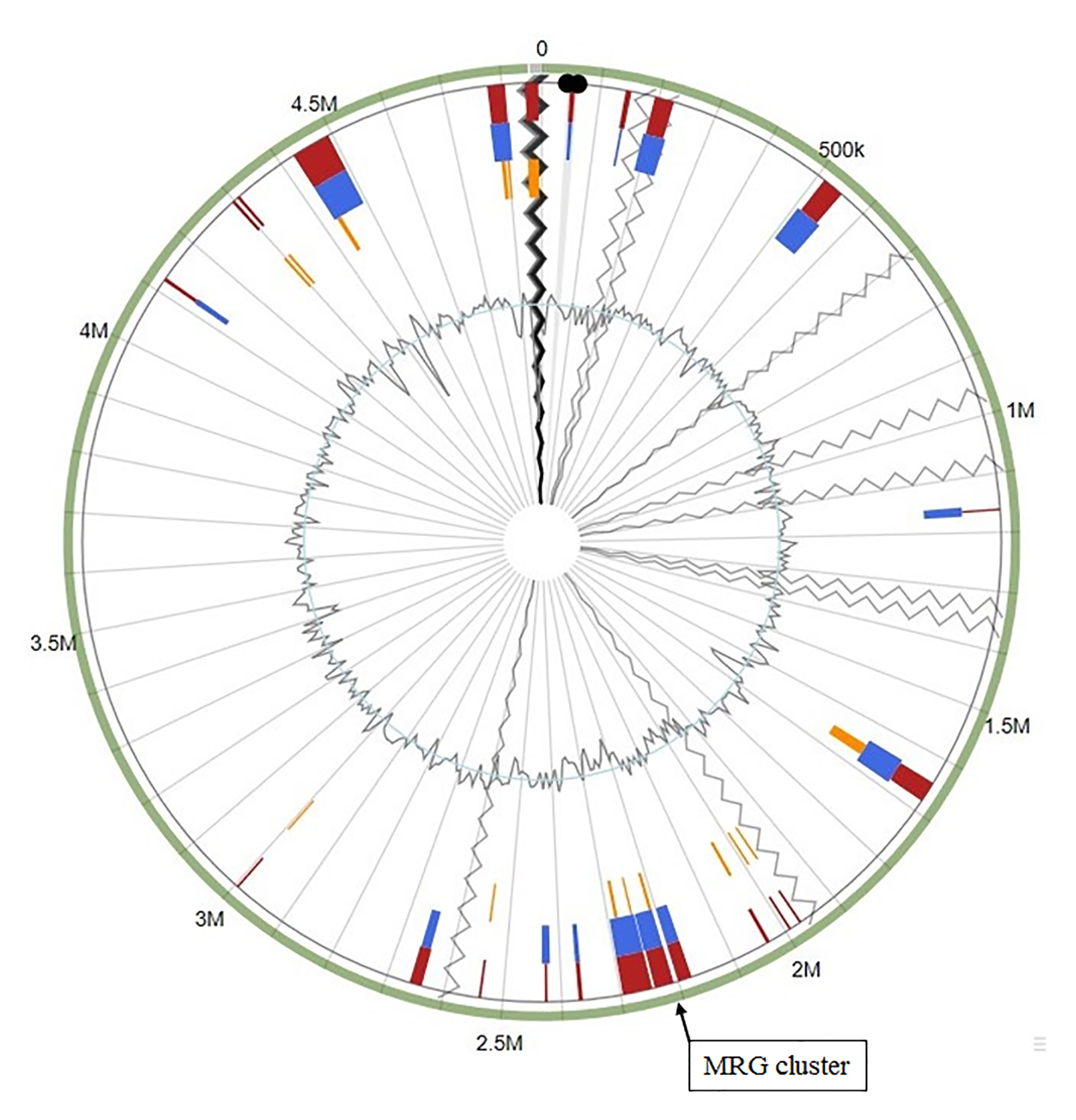

Supplement: Supplementary file 1 [file microorganisms-08-00153-s001.zip › Supplementary_material_Luziatelli/Figure S1_Luziatelli_et_al.tif]
