# Supplementary material for: Genome Sequencing of Pantoea agglomerans C1 Provides Insights into Molecular and Genetic Mechanisms of Plant Growth-Promotion and Tolerance to Heavy Metals
Source: Microorganisms. 2020 Jan 22;8(2):153. doi: 10.3390/microorganisms8020153 (PMC7074716; doi:10.3390/microorganisms8020153)
Supplement: Supplementary file 1 [file microorganisms-08-00153-s001.zip › Supplementary_material_Luziatelli/Table S1_Luziatelli_et_al_rev.doc]

**Table S1.** List of accession numbers for genomes utilized for phylogenetic analysis.

| **Strain** | **Species** | **Genome GenBank Accessions** |
| --- | --- | --- |
| C410P1 | *Pantoea agglomerans* | CP016889.1 |
| DSM3493^T^ | *Pantoea agglomerans* | FYAZ00000000.1 |
| IG1 | *Pantoea agglomerans* | BAEF00000000.1 |
| JM1 | *Pantoea agglomerans* | NHAS00000000.1 |
| LMAE-2 | *Pantoea agglomerans* | JWLQ00000000.1 |
| NCTC10500 | *Pantoea agglomerans* | UGSM01000000.1 |
| NCTC10601 | *Pantoea agglomerans* | UGSL01000000.1 |
| P5 | *Pantoea agglomerans* | NZ_NGNU00000000.1 |
| RIT273 | *Pantoea agglomerans* | JFOK00000000.1 |
| LMG 20103 | *Pantoea ananatis* | CP001875.1 |
| LMG 2665 | *Pantoea ananatis* | JFZU00000000.1 |
| PA13 | *Pantoea ananatis* | CP003086.1 |
| PNA 07-10 | *Pantoea ananatis* | QTTO01000000.1 |
| RIT 413 | *Pantoea dispersa* | QBJB01000000.1 |
| LMG 5346 | *Pantoea eucrina* | MIPP00000000.1 |
| Russ | *Pantoea eucrina* | MAYN01000000.1 |
| 3_1284 | *Pantoea* sp. | QNVM01000000.1 |
| AV62 | *Pantoea* sp. | NHBE00000000.1 |
| UBA3041 | *Pantoea* sp. | DFDP01000000.1 |
| UBA4549 | *Pantoea* sp. | DGLH01000000.1 |
| LMG 2632 | *Pantoea stewartii* subsp. *indologenes* | JPKO00000000.1 |
| DC283 | *Pantoea stewartii* subsp. *stewartii* | CP017581.1 |
| C9-1 | *Pantoea vagans* | CP002206.1 |
| MP7 | *Pantoea vagans* | JPKP00000000.1 |
| ZBG6 | *Pantoea vagans* | LFQL01000000.1 |
